# Antenatal Care Initiation Among Pregnant Women in the United Arab Emirates: The Mutaba'ah Study

**DOI:** 10.3389/fpubh.2020.00211

**Published:** 2020-06-11

**Authors:** Nasloon Ali, Iffat Elbarazi, Souha Alabboud, Fatima Al-Maskari, Tom Loney, Luai A. Ahmed

**Affiliations:** ^1^Institute of Public Health, College of Medicine and Health Sciences, United Arab Emirates University, Al Ain, United Arab Emirates; ^2^College of Health Sciences, Abu Dhabi University, Abu Dhabi, United Arab Emirates; ^3^Zayed Center for Health Sciences, United Arab Emirates University, Al Ain, United Arab Emirates; ^4^College of Medicine, Mohammed Bin Rashid University of Medicine and Health Sciences, Dubai, United Arab Emirates

**Keywords:** birth, cohort, infertility, mother, pregnancy, pregnancy trimesters, prenatal care, United Arab Emirates

## Abstract

**Introduction:** Antenatal care (ANC) provides monitoring and regular follow-up of maternal and fetal health during pregnancy. Women with appropriate ANC tend to have better delivery and birth outcomes. This study describes the patterns of ANC utilization and factors associated with appropriate ANC initiation in the United Arab Emirates (UAE) for the first time.

**Methods:** Baseline cross-sectional data from pregnant women who participated in the Mutaba'ah—Mother and Child Health Study between May 2017 and January 2019 was analyzed. Participants were recruited during ANC visits and completed a self-administered questionnaire that collected socio-demographic and pregnancy-related information and assessed whether it was their first ANC appointment. Regression models assessed the relationship between socio-demographic and pregnancy-related variables and “appropriate” (≤ 4 months' gestation) vs. “late” ANC initiation (>4 months' gestation).

**Results:** At recruitment, 841 participants reported that it was their first ANC visit and half (50.2%) of these women were late initiating their ANC. Mothers who were more educated, had previous infertility treatment or previous miscarriages were all more likely to achieve appropriate ANC initiation [adjusted odds ratio (aOR): 1.66, 95% confidence interval (CI): 1.05–2.62; aOR: 3.68, 95% CI: 1.50–9.04; aOR: 1.80, 95% CI: 1.16–2.79, respectively]. Women worrying about childbirth were less likely to achieve appropriate ANC initiation (aOR: 0.54, 95% CI: 0.34–0.85).

**Conclusion:** Half of pregnant women in this study did not achieve the global consensus guidelines on appropriate ANC initiation. Interventions among less educated women and those with previous pregnancy complications and childbirth anxiety are recommended to ensure appropriate ANC initiation.

## Introduction

Antenatal care (ANC) is the care provided by skilled healthcare professionals to women throughout their pregnancy. It includes risk identification and screening, prevention and management of pregnancy-related or concurrent diseases, and health education and promotion (1). Global guidelines recommend frequent medical care visits during the antenatal period to decrease the risk of maternal and perinatal mortality (1–3). Women are recommended to initiate their ANC appropriately during pregnancy as it will help to reduce complications, provide wider platforms for a healthier pregnancy, and maximize the benefits of monitoring fetal and maternal health (1). The World Health Organization (WHO) recommends that ANC should be initiated within the first trimester of gestation with at least four, and optimally eight visits during the pregnancy (1). Specifically, the WHO advises pregnant women to initiate contact during the first 12 weeks' gestation, with subsequent contacts taking place at 20, 26, 30, 34, 36, 38, and 40 weeks' gestation (1).

Previous studies have defined “late initiation” as attending to ANC in the 13th week (in their second trimester) or later (4, 5). Epidemiological studies have reported an association between the number of prenatal visits or gestational age at ANC initiation and pregnancy outcomes (6). Late ANC initiation may lead to poorer outcomes, such as low birth weight and preterm birth (7), and increases the total cost of prenatal care (8). Previous research has shown that late onset initiation of ANC may be more important than the frequency of visits on influencing outcomes (3, 6, 9, 10). Earlier work has reported that women with late ANC initiation are usually younger, higher parity or gravidity, without a partner, lower socioeconomic status, less educated, and have lower access to health services (5, 11, 12). Women who have unplanned pregnancies, recognize their pregnancy late, and had no complications in previous pregnancies will also tend to initiate ANC at later stages of their pregnancy (12). Moreover, women will not attend ANC if the quality of services is perceived as poor or their previous experiences were negative (13).

There has not been a systematic evaluation of the timing of ANC initiation, or the factors associated with late ANC initiation in the United Arab Emirates (UAE). This study will provide the first estimates on the patterns of appropriate utilization of ANC in the UAE and explore the associations between various socio-demographic and pregnancy-related variables and appropriate (≤ 4 months' gestation) versus late ANC initiation (>4 months gestation).

## Materials and Methods

### Study Design, Setting, and Participants

This is a cross-sectional analysis of the baseline data from pregnant women who participated in the Mutaba'ah Mother and Child Health Study. Mutaba'ah (which means to “follow up” in Arabic) is a prospective cohort study that aims to systematically recruit 10,000 pregnant women from the Emirati population during their ANC visits at the three major health institutions in Al Ain, Abu Dhabi, UAE. All pregnant women from the Emirati population who are 18 years and above, pregnant, resident in Al Ain, able to provide informed consent, and their newborns are eligible to participate in the study. Women who were eligible were recruited via consecutive sampling. Upon recruitment, women completed two questionnaires and are followed up during pregnancy via medical records in the hospitals. Then, the mothers and their offspring are to be followed up until the child turns 16 years of age using questionnaires, medical record extractions, and interviews. The Mutaba'ah Study has been described in detail elsewhere (14).

Data for the current analysis was extracted from the short questionnaire (SQ) administered during the first point of contact with the participants recruited between 25th May 2017 and 31st January 2019. The SQ includes 67 questions collecting data on demographics, psychosocial factors, previous pregnancies, and behaviors during her current pregnancy. This manuscript has been written in line with Strengthening the Reporting of Observational studies in Epidemiology (STROBE) guidelines (15).

### Patient and Public Involvement

Patients and public were not involved in the design or conduct of this analysis. However, during recruitment, participants provided feedback on the best methods to follow them and their children up after delivery, and strategies to maximize cohort recruitment for the Mutaba'ah Study.

### Variables and Measurement

The demographic and pregnancy-related characteristics included maternal education, employment, and age, number of people living in the house; number of previous pregnancies (gravidity), number of children (parity), complications in previous pregnancies including low birth weight, miscarriages, stillbirth and preterm births, gestational age, pregnancy planning status, maternal and paternal smoking status, consanguinity, perceived social support, and childbirth anxiety. Education was defined as “*More than High School*” and “*Less than High School*.” Women who responded as “*Illiterate*,” “*Never Attended School*,” “*Primary*,” or “*Secondary*” were labeled as less than high school and those who had responded “*Vocational or Diploma*,” “*Bachelors*,” “*Masters*” or “*Doctorate*” were labeled as more than high school. Women were queried on their anxiety toward childbirth and the factor worrying about birth was labeled as a “*Yes*” if they answered “*Yes, quite a lot*” or “*Yes, sometimes*” and “*No*” if they had answered “*No, not at all*” or “*No, not much*.” Similarly, social support was coded as a “*Yes*” or “*No*” based on their response to the question, “*Do you feel that you have enough people in your life to count on when you need anything?*”. Responses were coded as “*Yes*” if the respondent answered “*Yes, enough*” and “*Yes, definitely enough*” and was labeled as a “*No*” if they respondent answered as “*No, not much*” or “*No, not at all*.” Initiation was determined using the question: “*Is this your first antenatal visit for this pregnancy?*” with the options being “*Yes*” or “*No*”. A new dichotomous variable “*Appropriate Initiation*” and “*Late Initiation*” was created based on international pregnancy guidelines (2) for appropriate ANC initiation: “*Appropriate Initiation*” comprised women who had their first ANC visit during or before the first 4 months of gestation (first trimester) and “*Late Initiation*” comprised women who had their first ANC visit after 4 months of gestation. As the participants reported gestational age in months, 4 months was used as the cut-off for appropriate initiation.

### Statistical Analyses

Descriptive statistics were performed to show and compare the distribution of characteristics of the study population by ANC initiation status. Continuous variables were presented as means and standard deviations, while categorical variables as counts and percentages. Student *t*-tests were used to determine differences between group means for continuous variables (e.g., maternal age) and Pearson Chi-square tests were used for categorical variables (e.g., maternal education). Univariate and multivariate regression models were used to quantify the association between the different sociodemographic and pregnancy-related variables and ANC initiation. Backward stepwise multivariate analyses were performed with a removal criterion of *p* ≥ 0.10. Crude and adjusted odds ratios (aOR) with 95% confidence intervals (CI) were calculated. Statistical analyses were performed using Stata 15.1 (Stata Corp, College Station, TX). A *p* ≤ 0.05 defined statistical significance.

## Results

A total of 3,755 women enrolled in the Mutaba'ah cohort between 25th May 2017 and 31st January 2019. Among these women, 3,652 (97.3%) answered the question on whether it was their first ANC visit. Non-responders (2.7%) to the ANC question were not significantly different to responders in terms of age, number of pregnancies, or gestational age (data not shown).

**Figure 1:**
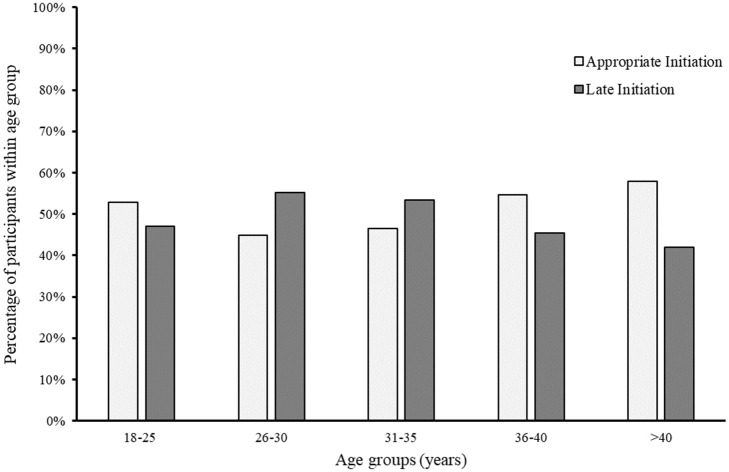
The distribution of ANC initiation categories (late and appropriate) among age groups in 841 pregnant women in Al Ain, UAE. The Mutaba'ah Study.

The women who answered “yes” to the cohort recruitment visit being their first antenatal appointment (*n* = 841, 23.0%) are the focus of this paper. Out of these 841 women, 422 women (50.2%) were classified as late for ANC initiation (more than 4 months' gestation). Figure 1 illustrates the distribution of ANC initiation categories (appropriate and late) within age groups. The socio-demographic and pregnancy-related characteristics of the study participants are described in Table 1. There were no significant differences in sociodemographic characteristics between the two groups of ANC initiation. The distributions of previous infertility treatment, previous pregnancy and birth complications, and childbirth anxiety were significantly different between women with appropriate and late ANC initiation (*p* ≤ 0.05). Six percent of those who initiated late reported previous infertility treatment as compared to 11.6% of appropriate initiators. Similarly, for women who had previous miscarriages, the distributions were 45.6 and 35.6% for appropriate initiation and late initiation, respectively (Table 1).

**Table 1:** Characteristics of 841 pregnant women according to whether they were classified with “appropriate” or “late” ANC initiation, in Al Ain, UAE.

	**Appropriate initiation (*n* = 419)**	**Late initiation (*n* = 422)**	***p***
Age (years)^†^	31.1.0 ± 6.2	30.6 ± 5.9	0.325
Gestational age (months)^†^	2.0 ± 0.8	6.4 ± 1.6	<0.001
Number of pregnancies^†^	3.8 ± 2.3	3.6 + 2.1	0.239
First child^*^			0.079
Yes	80 (44.4%)	100 (55.6%)	
No	335 (51.9%)	311 (48.1%)	
Planned pregnancy^*^			0.648
Yes	199 (50.5%)	195 (49.5%)	
No	199 (48.9%)	208 (51.1%)	
Worrying about birth^*^			0.032
Yes	250 (47.4%)	278 (52.7%)	
No	150 (55.4%)	121 (44.7%)	
Social support^*^			0.159
Yes	370 (51.1%)	354 (48.9%)	
No	31 (42.5%)	42 (57.5%)	
Education^*^			0.085
High school and below	246 (60.6%)	267 (66.4%)	
Diploma and above	160 (39.4%)	135 (33.6%)	
Employment^*^			0.375
Employed	121 (29.8%)	119 (29.7%)	
Unemployed/Student	285 (70.2%)	281 (70.3%)	
Number of people living in home^†^	11.8 ± 8.8	10.7 ± 9.0	0.098
Previous Gestational Diabetes Mellitus			0.851
Yes	91 (23.0%)	93 (23.5%)	
No	305 (77.0%)	302 (76.5%)	
Consanguinity^*^			0.125
Yes (via parents)	166 (46.8%)	147 (41.1%)	
No	189 (53.2%)	211 (58.9%)	
Previous infertility treatment^*^			0.005
Yes	46 (11.6%)	24 (6.00%)	
No	350 (88.4%)	376 (84.0%)	
Previous miscarriage^*^			0.011
Yes	145 (45.6%)	106 (35.6%)	
No	173 (54.4%)	192 (64.4%)	
Previous stillbirth^*^			0.017
Yes	25 (8.1%)	10 (3.5%)	
No	285 (91.9%)	277 (96.5%)	
Previous low birth weight baby^*^			0.068
Yes	142 (46.6%)	109 (39.1%)	
No	163 (53.4%)	170 (60.9%)	
Previous premature baby^*^			0.230
Yes	58 (18.4%)	43 (14.7%)	
No	258 (81.7%)	249 (85.3%)	

*The Mutaba'ah study*.

* *Missing data were excluded from the totals*.

† *Mean and standard deviation*.

Table 2 shows the associations between several self-reported socio-demographic and pregnancy-related factors and ANC initiation from univariate logistic regression models. Women with previous birth complications such as miscarriages [odds ratio (OR): 1.52, 95% CI 1.10–2.10] and stillbirths (OR: 2.43, 95% CI 1.15–5.15), as well as women who had previous infertility treatment (OR: 2.06, 95% CI: 1.23–3.44) were more likely to initiate appropriately (Table 2). Women who worried about giving birth were less likely to initiate on time (OR: 0.73, 95% CI: 0.54–0.97). Other variables commonly associated with late ANC initiation in the extant scientific literature, such as maternal age, number of pregnancies, household occupancy, and employment, did not reach the significance level in the univariate logistic regression models. In the multivariate model (Table 3), women who were more educated, had previous infertility treatment, or a miscarriage were more likely to initiate ANC appropriately (aOR: 1.66 (95% CI: 1.04–2.62), 3.68 (95% CI: 1.50–9.04), and 1.80 (95% CI: 1.16–2.79), respectively). Women who worried about giving birth were 46% (aOR: 0.54, 95% C1: 0.34–0.85) less likely to report appropriate ANC initiation.

**Table 2:** Univariate associations between self-reported socio-demographic and pregnancy-related factors and appropriate antenatal care initiation amongst pregnant women in Al Ain, UAE.

	**Unadjusted odds ratio (95% confidence interval)**
Age	1.01 (0.99–1.04)
Education	1.29 (0.97–1.71)
Employment	1.00 (0.74–1.36)
Number of pregnancies	1.04 (0.97–1.12)
Planned pregnancy	1.07 (0.81–1.41)
Consanguinity	1.26 (0.94–1.70)
Social support	1.42 (0.87–2.30)
Worry about birth	0.73 (0.54–0.97)
Previous infertility treatment	2.06 (1.23–3.44)
Previous miscarriage	1.52 (1.10–2.10)
Previous stillbirth	2.43 (1.15–5.15)
Previous low birth weight baby	1.36 (0.98–1.89)
Previous preterm baby	1.30 (0.85–2.00)

*The Mutaba'ah study*.

**Table 3:** Independent associations between self-reported socio-demographic and pregnancy-related factors and appropriate antenatal care initiation amongst pregnant women in Al Ain, UAE.

	Adjusted^*^ odds ratio (95% confidence interval)
Education	1.66 (1.05–2.62)
Previous infertility treatment	3.68 (1.50–9.04)
Previous miscarriage	1.80 (1.16–2.79)
Worry about birth	0.54 (0.34–0.85)

*The Mutaba'ah study*.

* *Model adjusted for age, education, employment, previous birth complications, consanguinity, number of pregnancies, infertility treatment, social support, worrying about birth, and planned pregnancy*.

## Discussion

The baseline findings from this large population-based study provide novel insights into the patterns of women's ANC initiation in the UAE. The study found that women in this population tended to initiate their ANC visits appropriately when they were more educated, had complications with previous pregnancies, and had previous fertility treatment. On the contrary, women with childbirth anxiety tended to initiate their visits later during pregnancy. Approximately 50% of study participants initiated ANC after the first 4 months of their pregnancy. In the UAE, like elsewhere in the world, pregnant women are encouraged to initiate their ANC before the end of the 13th week (i.e., third month) of pregnancy (16). In 2017, the UAE Ministry of Health and Prevention reported that Emirati women in the UAE had 99.1% ANC coverage (minimum one ANC visit) and 97.8% attended at least three ANC visits during their pregnancy (17). Although the UAE population might be meeting the appropriate numbers in terms of visits, the study findings suggest a gap in the appropriate timing of ANC. Amongst the late ANC initiation group in this study, the average month of initiation was six, which is the end of the second trimester. Health issues such as gestational diabetes mellitus (GDM), anemia and pre-eclampsia are often diagnosed during the standard testing practices in the second trimester (18). If women only begin their ANC visits at 6 months, it is unlikely that timely and effective interventions for the above health issues will be provided before they cause adverse health outcomes in the mother or the child. One study has suggested that GDM manifests even earlier in pregnancy and should therefore be tested earlier than the standard practice (19).

This study did not find any association between socio-demographic factors, except education, and ANC initiation. Educated pregnant women were 66% more likely to achieve appropriate initiation compared to less educated women. Educated women have consistently been found to have better health-seeking behaviors and are further empowered to seek and use health information (20, 21). This leads them to appreciate the importance of well-timed antenatal care (20, 21). As educational level has shown to be independently associated with appropriate antenatal care initiation, it is pertinent to empower women with a lower educational status to initiate ANC as appropriately as possible. Past studies have also shown that educational level is linked to lower morbidity and better lifestyle factors (22). To ensure more favorable pregnancy and birth outcomes, appropriate ANC initiation must be promoted to sub-populations of pregnant women who may be less educated.

This study shows a significant association between previous infertility treatment and an increased likelihood for appropriate ANC initiation. Nevertheless, one third of women with a history of infertility treatment still reported late ANC initiation. Appropriate prenatal screening and counseling are of utmost importance for pregnant women with a history of infertility. Women suffering from infertility are often older, (23) overweight, (24) or have chronic conditions such as thyroid dysfunction (25). These pregnant women may also have a poor prognosis in terms of outcomes as sub-fertile women, even after adjusting for age and parity, can suffer from pre-eclampsia, placenta previa, and placental abruption, and are more likely to experience induction of labor and undergo cesarean section (26). Sub-fertile women are also at an increased risk of preterm delivery, low birth weight babies, and spontaneous pregnancy loss (27). Delays in initiating ANC can increase all of the above poor outcomes especially for a high-risk population such as women with infertility issues (28). Hence, it is vital that most of the women with infertility history were initiating appropriately.

Women with previous miscarriages have been found to have better health-seeking behaviors in many other populations (29–34). The Mutaba'ah participants also seem to be on time for their ANC visits if they had previously experienced miscarriages. Appropriate ANC initiation is important for health providers as it allows for better monitoring of the fetus at an earlier stage as well as providing appropriate assistance for women with recurrent pregnancy losses (30).

Women anxious about childbirth were more likely to initiate late in the study population. Women who were worried about childbirth have been shown to have less positive expectations about their pregnancy and childbirth (29). Consequently, this can lead to negative associations between childbirth and fear of attending ANC which might further perpetuate their childbirth anxiety. Previous research examining the relationship between ANC and birth expectations found that negative expectations can lead to fear and this might result in tension and pain during labor (35). Antenatal care can enable women to develop the coping skills to replace negative attitudes with positive expectations toward labor and childbirth.

This study provides the first estimates of ANC initiation in a large representative sample of pregnant women in the UAE. In order to minimize selection bias, the study team aimed to recruit a representative sample of the population of pregnant women by using the main public and private hospitals as the recruitment sites with multiple recruiters at each location staggered throughout clinic hours. All of the Emirati population have full health insurance coverage providing them with the same level of health care at any health facility. As such, there is no difference in healthcare access between pregnant women attending these three hospitals and those who use other institutions. Therefore, a representative sample of the Emirati population in Al Ain can be recruited from these three hospitals. Furthermore, consecutive sampling of every eligible woman who attended the clinic allowed for the maximum number of participants to be recruited. However, the main limitation of this study is the lack of temporality associated with the cross-sectional design. That said, the reported associations in line with the extant scientific literature seem to rebut this possibility. The longitudinal design of the Mutaba'ah prospective cohort study will enable us to explore the direction and temporality of these exposure-outcome relationships and assess pregnancy and birth outcomes (e.g., GDM, preeclampsia, and low birth weight) among mothers with different ANC initiation timings in future studies. Moreover, qualitative studies nested within the main cohort may be useful to explore the sociocultural drivers related to late ANC initiation in this study's sample. The use of 4 months as a cut-off for appropriate initiation has been used elsewhere (36) and it was believed that it provided better estimates as the participants reported their gestational age in months.

In conclusion, half of the study sample did not achieve the global consensus guidelines on ANC initiation and many pregnant women delayed ANC initiation until their third trimester. Level of education, history of previous infertility or previous exposure to miscarriages, and pregnancy-related anxiety were independently associated with ANC initiation. Appropriate interventions could be offered to ensure that women without previous pregnancy complications and those with childbirth anxiety initiate antenatal care earlier in their pregnancy.

## Data Availability Statement

The data that support the findings of this study could be available from the Mutaba'ah Study.

## Ethics Statement

The study was approved by the United Arab Emirates University Human Research Ethics Committee (ERH-2017-5512), Al Ain Hospital Research Ethics Committee (AAHEC-03-17-058) and Tawam Hospital Research Ethics Committee (IRR−494). Informed written consent is obtained from the participant prior to the data collection.

## Author Contributions

LA and TL conceived, designed, and initiated the study. LA, TL, FA-M, IE, NA, and SA contributed to the planning of the study. LA, NA, IE, TL, FA-M, and SA contributed to the implementation, coordination, and management of the study. NA, TL, and LA drafted this manuscript. All authors read and approved the final version of the manuscript.

## Conflict of Interest

The authors declare that the research was conducted in the absence of any commercial or financial relationships that could be construed as a potential conflict of interest.
